# Sensory processing associated with subcategories of restricted and repetitive behaviors in Japanese children and adolescents with autism spectrum disorder

**DOI:** 10.3389/frcha.2024.1411445

**Published:** 2024-08-01

**Authors:** Haruka Noda, Naoto Yoneda, Ken Kamogawa, Goro Tanaka, Masakazu Ide, Ryoichiro Iwanaga

**Affiliations:** ^1^ Department of Occupational Therapy Sciences, Nagasaki University Graduate School of Biomedical Sciences, Nagasaki, Japan; ^2^ LITALICO Inc., Tokyo, Japan; ^3^ Minds & Hopes of SeichiAi no Kai Foundation, Saga, Japan; ^4^ The Porannohiroba Child Development Support Center, Nagasaki, Japan; ^5^ Department of Rehabilitation for Brain Functions, Research Institute of National Rehabilitation Center for Persons with Disabilities, Saitama, Japan

**Keywords:** autism spectrum disorder, sensory, restricted and repetitive behavior, repetitive sensory and motor behavior, insistence on sameness

## Abstract

**Background:**

Restricted and repetitive behavior (RRB) is a core symptom of autism spectrum disorder (ASD). The structure of RRB subcategories and their relationship with atypical sensory processing in Japan are not well understood. This study examined subcategories of the RRB in Japanese children with ASD and explored their relationship with sensory processing.

**Methods:**

A total of 103 children and adolescents with ASD participated in this study, with more than 70% having a co-occurring intellectual disability. First, exploratory factor analysis of the RRB items of the Social Responsiveness Scale second edition (SRS-2) was conducted to identify RRB subcategories. Second, Spearman correlation and multiple regression analysis were run to examine relationships between the RRB subcategories of SRS-2 and subsections of the Short Sensory Profile.

**Results:**

Exploratory factor analysis indicated a two factors solution; repetitive sensory and motor behavior and insistence on sameness. Multiple regression analysis suggested that Movement Sensitivity and Auditory Filtering were associated with insistence on sameness. Furthermore, Underresponsive/Seeks Sensation, Visual/Auditory Sensitivity, and diagnosis of intellectual disabilities were associated with repetitive sensory and motor behavior.

**Conclusions:**

Findings indicate that RRB subcategories are differently related to sensory processing patterns in children with ASD. These results suggested that RRB subcategories are beneficial to consider the relationship between RRB and sensory processing.

## 1 Introduction

Autism spectrum disorder (ASD) is a neurodevelopmental condition consisting of differences in social communication styles and a preference for restricted and repetitive behavior (RRB) (1). Both core features are necessary for the diagnosis of ASD, but the impact and clinical symptoms are heterogeneous (2–4).

### 1.1 RRB subcategories

Several studies have suggested that RRBs can be categorized into two types: repetitive sensory and motor behaviors (RSM) and insistence on sameness (IS) (5, 6). Here, RSM includes repetitive motion, unusual sensory interests, and repetitive use of objects, while IS includes obsessions/rituals, narrow interests, and difficulty with changes in daily life (7). Some studies have pointed out that a three-factor structure including circumscribed interests (CI) is more stable (8–10). Turner proposed that repetitive motor behavior and self-injurious behavior can be classified as lower-order RRB, while IS and CI are higher-order RRB domains (11). Although there are multiple opinions on how to classify RRB, there is a common understanding regarding the existence of the subcategories RSM and IS.

Research findings on how sex, age, and intellectual functioning in the ASD phenotype relate to the expression and severity of different RRB domains have been debated. Sex differences appear to have been reported in relation to RRB (8, 12, 13), while some other studies have not found this relationship (14, 15). Uljarevic et al. reported no sex differences in the IS domain, whereas males were associated with higher severity of RSM (10). In some cases, RSM was reported to be negatively correlated with age and intellectual function (5, 16) while in others indicated no significant association (8, 13). It has been suggested that RSM tends to decrease from later childhood to adolescence, while IS remains stable from early childhood to adolescence (17). It has also been reported that individuals with ASD and intellectual disability exhibited high RMB scores, whereas those without intellectual disability exhibited high scores in IS and CI (18). A large sample study with a wide age range suggested that RRB decreases as age increases and that the relationship between subcategories and age exhibits different patterns (19). Due to the variability across study reports, it is important to examine these factors when investigating RRB. To address these potential age-related differences, we included both children and adolescents in our study.

While it has been consistently suggested that RRB can be classified into several subcategories, the effects of cultural context on the classification of these subcategories are unknown. Some studies have shown differences in ASD symptoms across cultures. A cross-cultural study comparing autistic traits across India, Japan, and the UK revealed that while there was significant overlap in the items most predictive of an autism diagnosis, certain items exhibited cultural differences (20). Cross-cultural research has suggested that cultural aspects may influence RRB items more than other symptoms of ASD (21). It also showed that Autism-Spectrum Quotient (AQ) score variances differed between the UK and Japan (22). Most previous studies on RRB subcategories have been conducted in English-speaking countries; therefore, it is necessary to examine how the RRB subcategories would classify in different cultures, such as Japan, when we consider RRB subcategories.

No study has used factor analysis to identify RRB subcategories in Japanese samples of children with ASD. The Repetitive Behavior Scale-Revised (RBS-R) Japanese version adopted the same six subcategories as the original version (23) without factor analysis (24). In the Japanese version of the Social Responsiveness Scale, second edition (SRS-2), the RRB is treated as one of five subcategories, even though social communication and interaction are divided into four patterns. In this study, we will attempt to identify the RRB subcategories of Japanese children with ASD by examining the exploratory factor structure of the RRB items of SRS-2, which are frequently used to measure ASD symptoms.

### 1.2 Association between RRB subcategories and sensory processing problems

For understanding ASD symptoms and RRB, sensory processing problems are an important topic. Sensory processing problems in people with ASD have been frequently reported (25, 26). Different aspects of ASD symptoms are associated differently with various patterns of sensory processing. In the correlation analysis of the AQ (27) and the Adult/Adolescent Sensory Profile (AASP) (28), attention to detail and imagination were not significantly correlated with the total score of AASP; social skills, attention switching, and communication were significantly correlated with the total score of AASP (29). Some sensory subtype studies by Lane et al. showed sensory subtypes classified according to modality and responsiveness rather than sensory severity (30–32). Thus, the relationship between sensory processing characteristics and ASD symptoms does not simply increase with severity but rather appears to be heterogeneous in individuals with ASD.

Although several studies have suggested that RRB is associated with sensory processing, there is no consensus on whether these behavioral categories are conceptually distinct (33, 34). Unusual sensory responses and RRB are included in the same group of ASD as different behavioral types in the DSM-5 (1). Furthermore, RRB was associated with sensory characteristics even when adjusted for age and IQ (35, 36). An exploratory model has proposed that atypical sensory function is a significant mediator of RRB through intolerance of uncertainty (37). Schulz et al. showed that sensory hypersensitivity predicted repetitive behaviors in both typically developing (TD) groups and ASD groups (38). In a study analyzing video recordings, hyperresponsive behaviors were more associated with everyday sensory stimuli and family-initiated stimuli (e.g., TV sound, sunlight) and everyday actions (e.g., brushing teeth, washing face), while sensory seeking was more associated with free play and child-initiated stimuli (39). Sensory over-responsivity and high RRB were significantly associated with internalizing symptoms (40). These results suggest that a unidirectional or reciprocal relationship between RRB symptoms and sensory processing exists.

It has been suggested that each of the RRB subcategories has a different relationship with sensory processing characteristics. In a study using the Repetitive Behavior Questionnaire-2 and ASSP, IS was significantly correlated with all types of responsiveness, and RSM was significantly correlated with Avoidance/Seeking (6). Another study showed that sensory sensitivity predicts RRB, regardless of the ASD diagnosis (38). In this study, repetitive motor movements or rigidity and adherence to routine had a small association with RRB, and preoccupation with restricted patterns of interest and unusual sensory interests were predictors. However, the associations between RRB subcategories and each sensory modality are unclear.

### 1.3 Research questions

There are two research questions in the present study. The first research question was to determine what structure was appropriate for RRB subcategories in children and adolescents with ASD in Japan. We hypothesized that RRB would be divided into two subcategories (RSM, IS) as in previous studies (5, 6, 41, 42). The second research question was to investigate how the RRB subcategories relate to sensory processing in children and adolescents with ASD. Our hypothesis was that the RRB subcategories might be associated with sensory processing properties in different patterns.

## 2 Methods

### 2.1 Participants

Since all participants were minors, we obtained written informed consent from all the parents/caregivers of the participants, in accordance with the Declaration of Helsinki. The present study obtained approval from the Ethics Committee of the Graduate School of Biomedical Sciences at Nagasaki University (17110906).

Our study included 103 children with ASD aged 6–18 years (*mean ± SD* = 12.8 ± 4.0). Among them, eighty-one participants (78.6%) were male. They were recruited from autism parent associations across various prefectures and from day-service centers for children with developmental disorders in Japan. Caregivers provided reports on their children's neurodevelopmental disorder diagnoses from medical institutions. All participants had received a diagnosis of ASD (including autism and Asperger syndrome). The average total T-score on the Social Responsiveness Scale, Second Edition, which indicates the severity of autism symptoms, was 84.7 (*SD* = 14.8). Additionally, 71.8% of participants were diagnosed with intellectual disabilities. Children with genetic disorders (e.g., Fragile X, Down syndrome) or known medical conditions such as cerebral palsy, epilepsy, or brain injury were excluded from the study.

### 2.2 Materials

#### 2.2.1 Short Sensory Profile (SSP)

The SSP is a 38-item parent-reported questionnaire for assessing sensory processing characteristics (43). The SSP is organized into seven subsections: Tactile Sensitivity, Taste/Smell Sensitivity, Movement Sensitivity, Underresponsive/Seeks Sensation, Auditory Filtering, Low Energy/Weak, and Visual/Auditory Sensitivity. Parents were asked to rate the frequency with which their child engages in behaviors related to sensory processing in each subsection. Possible scores ranged from 1 to 5, from “never” (1 point) to “always” (5 points). In the Japanese version of SSP, a lower score indicated typical sensory responses, while a higher score indicated atypical sensory responses, which is opposite to the original version of SSP (44). The Japanese version of the SSP also calculated standard scores of children ages 11–18 years, unlike the English version. In this study, the SSP total score and the raw score of each subsection are included in the analysis.

#### 2.2.2 Social Responsiveness Scale, second edition (SRS-2)

The SRS-2 is a standardized, parent-reported questionnaire used for screening ASD risk and assessing ASD symptoms (45). The Japanese version of the SRS-2 School-Age Form, which was standardized for ages 4–18 years, was utilized in this research (46). It includes 65 items about children's behavior over the past six months. In the Japanese version of SRS-2, caregivers were required to respond 0 (Not True) to 3 (Almost Always True). Higher scores indicate more severe autism symptoms in each subdomain. The total raw score and each subdomain score of the SRS-2 can be converted to T-scores based on chronological age and sex norms. The symptomatology scores are classified into three categories: “Mild” (60–65), “Moderate” (66–75), and “Severe” (76 and above). In the present study, participants with a total T-score of less than 60 on the SRS-2 were excluded; however, no samples were excluded because all the collected data had T-scores of 60 or above. All items are clustered into a two-factor structure based on DSM-5 diagnostic criteria, which includes five subdomains corresponding to these two factors; social communication and interaction (social awareness, social cognition, social communication, and social motivation) and restricted, repetitive behaviors and interests (12 items).

### 2.3 Statistical analyses

#### 2.3.1 Research question 1

Exploratory factor analysis (EFA) was conducted on RRB items of SRS-2. The EFA was carried out using the minimum residual solution with Promax rotation. Parallel analysis, which is based on the Monte Carlo simulation (47), and minimum average partial (MAP) were used to determine the number of factors, which have been proposed as suggested criteria (48). Factor loadings for items were set at .30. EFA was based on the raw scores of RRB items of SRS-2, and was performed using the GPA rotation (49) and psych (50) packages in R (v4.3.3, Vienna, Austria).

#### 2.3.2 Research question 2

Spearman correlation coefficients were calculated for the raw scores of the subscales of the SRS-2 (including IS and RSM), SRS-2-total T-score, and the SSP, to examine the relationship between detailed sensory processing features and ASD symptoms. To account for multiple comparisons, correlation results are presented with Bonferroni adjusted alpha levels. Next, we conducted a two-step hierarchical linear regression to investigate SSP subscales that explained the unique variances of RRB subscales. These analyses were performed using the corrplot (51) and stats (52) in R (v4.3.3, Vienna, Austria).

## 3 Results

### 3.1 Research question 1

The Kaiser–Meyer–Olkin measure of sampling adequacy was .80, and Bartlett's test of sphericity was significant (*p* < .001, approximate *χ^2^ *= 429.23).

The results of the parallel analysis showed that the eigenvalues after the second factor were smaller than those from the simulation, indicating that a two-factor solution should be retained in the final analysis. Detailed results of the parallel analysis are shown in Supplementary Table S1. The MAP also indicated the two-factor structure was appropriate, therefore the two-factor structure was selected for the EFA.

Table 1 shows the final two-factor solution, which accounted for 38.0% of the variance (RSM 24.4%, IS 13.6%). All 12 items in the RRB factor of SRS-2 were included in the factor analysis, and no items were loaded on two factors.

**Table 1:** Factor structure on the RRB items of SRS-2.

	Item No.	Summaries of items	Factor loading	
			Factor 1	Factor 2
Factor 1: Repetitive sensory motor behavior				
	SRS20	Unusual sensory interests and playing with toys in unusual ways	.**84**	−0.20
	SRS50	Repetitive body movement	.**84**	−0.08
	SRS8	Strange behavior	.**76**	.11
	SRS63	Strange touch to people	.**66**	−0.07
	SRS29	Being thought strange or weird	.**58**	.29
Factor 2: Insistence on sameness				
	SRS24	Difficulties in changing routine	.24	.**58**
	SRS4	Stubborn under stress	.05	.**57**
	SRS31	Thinking about one thing	−0.19	.**45**
	SRS14	Difficulty with physical activities	−0.10	.**44**
	SRS28	Thinking and talking about the same things	.01	.**41**
	SRS39	Narrow range of interests	.18	.**39**
	SRS49	Can do very limited things well	.02	.**30**
				
Factor correlation				
	Factor 1		1	.342
	Factor 2		.342	1

Factor loadings greater than 0.3 are bolded to highlight significant loadings.

### 3.2 Research question 2

#### 3.2.1 Correlation

Spearman's correlation analyses were conducted to examine the relationships between RRB subcategories and SSP subsections. Significant correlations were shown between RSM and Underresponsive/Seeks Sensation (*r* = . 527, *p* < .001), Visual/Auditory Sensitivity (*r* = .399, *p* < .01); IS and Movement sensitivity (*r* = .405, *p* > .01), Underresponsive/Seeks Sensation (*r* = .416, *p* < .001), Auditory Filtering (*r* = .399, *p* > .001), and Low Energy/Weak (*r* = .441, *p* < .001), respectively (Table 2).

**Table 2:** Spearman correlation between RRB subcategories and SSP subsections.

	TAC	TSM	MOV	USS	AFL	LEW	VAS
RSM	.113	−0.033	.100	.527**	.305	.039	.399*
IS	.288	.228	.405*	.416**	.541**	.441**	.294

RSM, repetitive sensory and motor behaviors; IS, insistence on sameness; TAC, tactile sensitivity; TSM, taste/smell sensitivity; MOV, movement sensitivity; USS, underresponsive/seeks sensation; AFL, auditory filtering; LEW, low energy/weak; VAS, visual/auditory sensitivity.

* *p *< 0.0.

** *p* < 0.001.

#### 3.2.2 Multiple regression analysis

In the hierarchical regression predicting IS, the base model with demographic variables was not a significant predictor, but intellectual disability was a significant predictor of RSM (Table 3). In Model 2, SSP subsections were added as a predictor. Movement Sensitivity and Auditory Filtering were significant predictors of IS [*R^2^* = .4525, *p* < .001, *F* (10, 92) = 7.605]. In the model of RSM, Underresponsive/Seeks Sensation (*p* < .001), Visual/Auditory Sensitivity (*p *< .01), and Intellectual disabilities (*p* < .01) were significant predictors [*R^2^* = 0.3974, *p* < .001, *F* (10, 92) = 6.067].

**Table 3:** Hierarchical multiple regression analysis between RRB subcategories and SSP subsections.

			RSM				IS			
Predictor			R2	*Δ*R2	*β*	*F*	R2	*Δ*R2	*β*	*F*
Model 1			.11			3.95**	.03			.99
	Age				.03				.06	.03
	Sex				.25				−1.26	.25
	Diagnosis of ID				2.99**				.49	2.99
Model 2			.40	.29		6.07***	.45	.42		7.61***
	Age				.08				.07	
	Sex				−0.40				−1.51	
	Diagnosis of ID				2.05*				−0.16	
	SSP subsections									
		TAC			−0.08				.00	
		TSM			−0.01				.07	
		MOV			.12				.40**	
		USS			.31***				.10	
		AFL			.04				.23**	
		LEW			−0.12				.08	
		VAS			.22*				.01	

RSM, repetitive sensory and motor behaviors; IS, insistence on sameness; ID, intellectual disabilities; SSP, short sensory profile; TAC, tactile sensitivity; TSM, taste/smell sensitivity; MOV, movement sensitivity; USS, underresponsive/seeks sensation; AFL, auditory filtering; LEW, low energy/weak; VAS, visual/auditory sensitivity.

* *p *< .05.

** *p* < .01.

*** *p* < .001.

## 4 Discussion

The purpose of our study was to identify RRB subcategories and examine their relationship to sensory processing patterns. RRB items of the SRS-2 were structured into a two-factor solution similar to previous studies: RSM and IS, which were strongly associated with different sensory processing characteristics.

### 4.1 Research question 1

The result of the EFA showed that RRB items of the SRS-2 were explained by the two-factor structure; RSM and IS. This two-factor structure is similar to past research (5, 7). Although there are studies suggesting that RRB items may be affected by culture (21), the present study may imply that easily noticeable symptoms may vary by culture but may not significantly affect the structure of the RRB subgroups.

Some studies have suggested that a three-factor solution that includes CI is appropriate (8, 9) and some others proposed more factors (18, 23). In this regard, the bias of the concepts measured by the RRB items of the SRS-2 and the small number of items may have influenced the results. However, some studies that support more than three factors also agree on the existence of the RSM and IS factors (8, 9, 18). Although the number of subcategories is a subject of debate in previous studies (5, 8, 10), our analysis showed a two-factor structure was appropriate for the RRB items of the SRS-2. Therefore, the present data suggest that the RRB of SRS-2 can be classified into two subgroups (RSM and IS) at least in Japanese children and adolescents with ASD.

### 4.2 Research question 2

Research Question 2 examined what sensory processing characteristics are strongly associated with each RRB subcategory. The results showed that significant predictors differed between RSM and IS, providing new evidence for the existing literature reporting the association between RRBs and sensory processing (6, 38, 53).

For Underresponsive/Seeks Sensation and Visual/Auditory Sensitivity, RSM exhibited significant correlations and was a significant predictor in the hierarchical regression analysis. As a clinical implication, RSM may function as a compensatory behavior. An association between vestibular stimulation and low-order RRB has been reported; Gal and Dyck (54) found that blind children were more likely than low-vision children to engage in rocking and repetitive head movements, which are proprioceptive and vestibular-seeking behavior. This suggested that they may function as compensatory behaviors in situations lacking sensory input, and it supports our finding that Underresponsive/Seeks Sensation is associated with RSM. On the other hand, there are several studies supporting a relationship between RSM and hypersensitivity in the visual or auditory modality (38, 55), which is consistent with our findings. These results suggest that some IS behaviors may be compensatory behaviors triggered by discomfort due to atypical visual and auditory functions.

On the other hand, it is debatable whether it was appropriate to treat sensory reactivity collectively as the Underresponsive/Seeks Sensation subsection in the SSP. Boyd et al. suggested a significant association between sensory seeking and ritualistic/sameness (36), which is similar to the present study. Lidstone et al. suggested RSM was significantly correlated with seeking, but not with low registration (6). While Underresponsive/Seeks Sensation was higher in the ASD group than the TD group (26), it also showed a negative correlation between AQ and low registration and a positive correlation with seeking (29). Given the variability in research findings regarding the relationship between ASD symptoms and response characteristics, it may be a more appropriate way to separate Underresponsive/Seeks Sensation as hyposensitivity and seeking. This is a limitation caused by the factor construction of SSP.

IS showed that significant positive correlations were found with Movement Sensitivity, Underresponsive/Seeks Sensation, Auditory Filtering, and Low Energy/Weak. Furthermore, hierarchical regression analysis showed that IS was significantly predicted by Movement Sensitivity and Auditory Filtering. Movement Sensitivity was an SSP subsection with a small effect size compared to ASD and TD (26) and not a significant difference compared to developmental delays (56), but the results of the present study suggested that it significantly predicts IS. Both IS and RSM have been shown to correlate with the center of pressure sway area during a quiet stance (57). However, the Movement Sensitivity of the SSP consists of items that measure mainly vestibular anxiety or sensitivity and not simple postural control. Therefore, it may be the vestibular anxiety part that is more closely related to IS rather than the ability to maintain posture. It has been suggested that IS, not RSM, is associated with anxiety. The relationship between IS and anxiety is mediated by sensory avoidance/hypersensitivity (6) and is partially mediated by social motivation, not RSM (58). Given the notion that repetitive behaviors may work as actions to gain reassurance through sensory input (33), Movement Sensitivity may be more likely to increase heightened anxiety in daily life, and consequently IS behaviors also increase to reassure themselves. Tomchek et al. demonstrated in their SSP study that Auditory Filtering is significantly higher in ASD than TD (26). This subsection includes difficulty noticing social information and distractibility of sounds, indicating a tendency of disruption by an auditory stimulus. An examination of auditory modalities showed a significant correlation (*r* = −0.37 in the ASD group) between overall RRB and auditory sensitivity, whereas auditory sensitivity was not a predictor (6, 38, 53). In the present study, Visual/Auditory Sensitivity was one of the significant predictors of RSM. Although the association between auditory modality and RRB cannot be ruled out, differences in reactivity, such as distractibility and hypersensitivity within the modality, may each be associated with different aspects of RRB.

It was also shown that the diagnosis of intellectual disability did not predict IS but significantly predicted RSM. Although the relationship between RRB and intellectual disability or cognitive functioning is debated, some studies support our findings. It has been reported that RSM was correlated with IQ and age, whereas IS was unrelated to these factors (5, 59). Individuals with ASD and intellectual disability have been reported to exhibit high scores in RSM (18). IQ score was a significant predictor of the total score on the RBS-R (38). Although the variability in previous research findings should be considered, the results of the present study suggest that RSM may be more strongly associated with intellectual aspects compared to IS.

### 4.3 Limitations

This study has several limitations. First, our results are based on parental responses. It is difficult to distinguish between hypo-sensitivity and hypo-reactivity based on observational scaling studies alone. The present study would be enhanced by incorporating directly observed data, such as ASD symptoms or experimental data on the physiology of sensory processing. Second, the reliability of the SSP scale: As Williams et al. point out, there are concerns about measuring sensory processing characteristics of individuals with ASD using the SSP factor (60). While similar concerns exist regarding constructs, the distributional bias in the ASD group due to the change to the Japanese version remains untested. In the future, it may be necessary to examine the reliability of the Japanese version of the SSP scale. Third, there may be sample bias. Although we were not able to measure the IQ of everyone in our sample, the proportion of children with intellectual disability was relatively high compared with prevalence studies (61, 62). The study results might be captured based on a sample with a relatively lower IQ. Therefore, it is desirable to verify these findings with a larger sample study. Fourth, the wide age range of the participants, from 6 to 18 years, is a limitation. This broad age range may obscure distinct characteristics of RRB that exist across narrower age bands. If distinct characteristics of RRB exist across narrower age bands, these differences might not be clear in the present study. Fifth, this study conducted EFA, not CFA. While it is desirable to perform the CFA following the EFA to statistically validate the factor structure, this study was unable to prepare a separate dataset for the CFA. Future research should report the fit indices from the CFA to confirm construct validity in more detail.

### 4.4 Implications and future direction

Although there are limitations, the findings from this study provide insights into the relationship between RRB and sensory processing patterns in Japanese children and adolescents with ASD. The finding that RSM and IS are related to sensory processing in different patterns suggests that dividing RRB into subcategories rather than treating it as a single entity may be more effective in identifying the underlying causes and developing better support strategies. These findings may help develop approaches for children with ASD. Our results may provide evidence that intervention strategies can be tailored based on whether clients exhibit different patterns of RSM or IS, and on the specific sensory processing characteristics they struggle with.

Future research should investigate two directions. First, it should explore the developmental trajectories of RRB subcategories and their relationship with sensory processing over time. Longitudinal studies can provide deeper insights into how these behaviors evolve and the long-term effectiveness of targeted interventions. Second, CFA should be applied to other datasets of the Japanese sample of with ASD. This analysis will provide more robust evidence for the validity of the two-factor structure examined in this study.

## 5 Conclusion

The present study suggests that the RRB subcategories are classified as RSM and IS in the Japanese sample, similar to previous studies. RSM and IS were associated with different sensory processing characteristics. This information is valuable for examining the common neural basis and reasons for the appearance of sensory processing characteristics and RRB subcategories.

## Data Availability

The raw data supporting the conclusions of this article will be made available by the authors, without undue reservation.
