# Supplementary material for: Sensory processing associated with subcategories of restricted and repetitive behaviors in Japanese children and adolescents with autism spectrum disorder
Source: Front Child Adolesc Psychiatry. 2024 Aug 1;3:1411445. doi: 10.3389/frcha.2024.1411445 (PMC11732166; doi:10.3389/frcha.2024.1411445)
Supplement: Supplementary file 1 [file Table1.docx]

Supplementary Material

## Supplementary Table

**Supplementary Table 1. Parallel analysis results to determine the number of factor**

| factor number | 1 | 2 | 3 | 4 | 5 |
| --- | --- | --- | --- | --- | --- |
| FA actual data | 3.69 | 1.30 | 0.37 | 0.18 | 0.11 |
| FA simulated data | 0.93 | 0.53 | 0.40 | 0.28 | 0.17 |

Note: FA = Factor Analysis. The table compares the eigenvalues from the actual data with those from the simulated data to determine the appropriate number of factors to retain. Eigenvalues greater than the corresponding simulated data indicate factors to be retained.
